# The (Mathematical) Modeling Process in Biosciences

**DOI:** 10.3389/fgene.2015.00354

**Published:** 2015-12-22

**Authors:** Nestor V. Torres, Guido Santos

**Affiliations:** ^1^Systems Biology and Mathematical Modelling Group, Departamento de Bioquímica, Microbiología, Biología Celular y Genética, Sección de Biología de la Facultad de Ciencias, Universidad de La LagunaSan Cristóbal de La Laguna, Spain; ^2^Instituto de Tecnología Biomédica, CIBICANSan Cristóbal de La Laguna, Spain

**Keywords:** biosciences, biological system, model, mathematical model, systems biology

## Abstract

In this communication, we introduce a general framework and discussion on the role of models and the modeling process in the field of biosciences. The objective is to sum up the common procedures during the formalization and analysis of a biological problem from the perspective of Systems Biology, which approaches the study of biological systems as a whole. We begin by presenting the definitions of (biological) system and model. Particular attention is given to the meaning of mathematical model within the context of biology. Then, we present the process of modeling and analysis of biological systems. Three stages are described in detail: conceptualization of the biological system into a model, mathematical formalization of the previous conceptual model and optimization and system management derived from the analysis of the mathematical model. All along this work the main features and shortcomings of the process are analyzed and a set of rules that could help in the task of modeling any biological system are presented. Special regard is given to the formative requirements and the interdisciplinary nature of this approach. We conclude with some general considerations on the challenges that modeling is posing to current biology.

## Introduction

*A theory has only the alternative of being right or wrong. A model has a third possibility: it may be right, but irrelevant*.

***Manfred Eigen. The Origins of Biological Information.***

There are many definitions of science (Popper, 1935; Kuhn, 1962, 1965; Lakatos, 1970), but all of them refer to a body of knowledge obtained through a particular method based on the observation of the physical world, linked to systematically structured reasoning, strategies by which general principles and laws are deduced. That particular method is the “Scientific Method”, defined by the Oxford English Dictionary as “*…the procedure…, consisting in systematic observation, measurement, and experiment, and the formulation, testing, and modification of hypotheses*.” In the above statements there are two core ideas which are relevant here and that derive directly from what science is: the first one is that any scientific activity requires measurements and thus, quantification of real magnitudes. The second is that any scientific activity makes sense only if it allows us to gain “knowledge”; that is understanding, predicting and control. In science these goals are achieved through the building of models and theories. Both serve, with different degrees of generality, to explain the observed facts and predict with high probability the evolution and behavior of natural systems.

### Biological Systems and Models

Before describing the modeling process, it is advisable to clarify the meaning of two key concepts, “biological system” and “model” that we assume are inextricably linked.

Any biological system is composed of a set of elements, physical objects, usually numerous and diverse, that influence each other (i.e., they interact) and that are physically and functionally separated from their environment. The physical separation is a frontier, which can be real (e.g., a membrane) or imaginary, which is permeable to matter, and energy (i.e., an open system). The functional separation is a consequence of the fact that biological systems are far from thermodynamic equilibrium, in contrast with the environment. The interchange of matter and energy with the environment is indeed a necessary requisite to sustain the chemical–physical processes that occur far from equilibrium. Thus defined, a living system involves a reference to the environment in which it is located and with which it interacts. It is worth noting here that when we focus solely on the elements, disregarding the interactions between them and with the environment, the system disappears, because a set of entities devoid of interaction is a mere aggregation of elements. This is the essence of “system”, a holistic approach to research as opposite to a reductionist view.

For our purposes here, a model is a conceptual or mathematical representation of a system that serves to understand and quantify it. The difference between conceptual and mathematical resides only on the way the representation is formulated. A model is always a simplified representation of the reference system, which the scientist wishes to understand and quantify. It ultimately serves as a means of systematizing the available knowledge and understanding of a given phenomenon and the facts concerning it.

A first step in any model-building attempt is the simple verbalization of statements about the biological system. Soon this phase leads to a more productive one, where observations and hypothesis transform the observations and data into an organized core, the so-called “conceptual” model. Conceptual models constitute, thus, a first level of qualitative integration of the information on the system under scrutiny. Conceptual models are so ingrained in our everyday life that we usually do not make a distinction between models and the real thing. Very often, they come as diagrams, words or physical structures, which deal with either the structure and/or the function of the real system. The causal diagrams are examples of suitable tools that help in dealing with the conceptual models (Voit, 1992; Minegishi and Thiel, 2000; Allender et al., 2015).

A key feature of the conceptual models is that they only make a qualitative description of the real system. Examples of such conceptual models in biology range from the typical plant or animal cell diagram (one that integrates many observations of multiple types of cells obtained through a great variety of techniques) to the models about enzyme action and metabolic pathways. The enzyme action model describes how the substrate attaches to the active site of the enzyme, and how the enzyme structure changes in different molecular environments. Another ubiquitous conceptual model is that of metabolic pathways; they represent the coordinated and sequential activities and regulatory features of many enzymes. The main value of the conceptual models is that, as the result of the (tough) complex process involved in its development, it allows the integration of disperse information obtained from different sources. However, their origin renders them imprecise, and conceptual models can be interpreted differently by different people.

A further refinement in the process of system understanding is given by the translation of the conceptual model into a form subject to a quantitative description, evaluation and validation. This form is the mathematical model. A mathematical model is the formalized description of the system derived from a previous conceptual model. Mathematical models may be very diverse in nature. Dynamical models consider changes in the elements with time, and can be categorized into deterministic and stochastic. In the deterministic ones, the velocities only depend on the concentration of the elements and the parameters of the model. The opposite are the stochastic ones, in which the velocities also depend on the random noise of the system, due to the uncertainty present in systems containing statistically non-abundant elements. On the other hand, static models try to understand the structure of the interconnection of the elements, which remains constant during time under specific conditions (Voit, 2012).

The mathematical models not only help us to understand the system, but also are instrumental to yield insight into the complex processes involved in biological systems by extracting the essential meaning of the hypotheses (Wimsatt, 1987; Bedau, 1999; Schank, 2008) and allows to study the effects of changes in its components and/or environmental conditions on the system’s behavior; that is, they allow the control and optimization of the system.

### Mathematical Models in Biology

The usefulness of mathematical models in physics and technology is well documented; in fact they can be traced back to the very origins of physics. Since the days of Galileo, Kepler and Newton scientists have striven to develop their models by means of mathematical formalism. What we want to present and develop here is the tenet that modeling in general, but specifically mathematical modeling, particularly in biology –as well as in science in general- is the only way to attain such quantitative understanding and control. Mathematical modeling should thus be an essential and inseparable part of any scientific endeavor in the realm of XXI century bioscience.

It has been claimed that the maturity of a scientific field correlates positively with how often mathematical models are developed and used to understand and control the real system (Weidlich, 2003; Medio, 2006; Brauer and Castillo-Chavez, 2010; Gunawardena, 2011). In this regard, it has not been until recently that dynamic mathematical models in biology have become a common feature. Besides the well-known cases of the Michaelis–Menten model to describe the dynamics of the enzyme-catalyzed reactions (Michaelis and Menten, 1913) and its subsequent development for the case of allosteric enzymes (Monod et al., 1965), the Hodgkin–Huxley model of the action potentials in neurons (Hodkin and Huxley, 1952), the Lotka–Volterra model about the interaction of species (Lotka, 1920; Volterra, 1926) and the epidemiological models of epidemics (Ross, 1915; MacDonald et al., 1968), the emergence and widespread recognition of the role and importance of mathematical models in biology is a recent phenomenon.

It is easy to understand why only until very late in scientific research mathematical modeling of biological systems has been put in use. Biological systems, by their nature, are refractory to precise quantitative and mathematical description. They are composed by many elements closely interconnected by processes and interactions that take place at different levels of organization (molecular, cellular, in tissue, whole animals and ecological). At the same time, these processes occur in an open system as a result of the existence of multiple gradients far from the thermodynamic equilibrium, which in the end produce very complicated non-linear dynamics between the elements of the system (Prigogine, 1961). This situation has impaired the quantitative and dynamic approach to the understanding of biological systems through the use of mathematical models.

However, two technological advancements that have made feasible the construction and resolution of mathematical models for biological systems have been developed in the last decades. There is a general accessibility and almost universal ubiquity of the computational power required for the management of information and the calculation of large systems. On the other hand, the development of the high throughput techniques and the emergence of the “omics” sciences (genomics, transcriptomics, proteomics, signalomics, and metabolomics) have generated a great deal of dynamic information on the structure and behavior of the biological systems. This information has become easier and cheaper to acquire, process and store than ever before.

All the above have been instrumental to the arrival of Systems Biology, as the XXI century approach to the quantitative and interdisciplinary study of the complex interactions and the collective behavior of a cell, an organism or an ecosystem. The distinctive feature of Systems Biology is the concern with the organization and biological function. This approach goes beyond the classical reductionist approach, where the researcher seeks to understand the systems by breaking them down into their constituent elements and analyzing them separately or, in a novel version of the old paradigm facilitated by the high throughput techniques, by collecting every piece of accessible information. In the Systems Biology approach, research is focussed not on the parts considered individually, but on the relationships that exist between the structural components of biological systems and their function, and on the characteristics of the interactions that occur between different sub-systems. This method allows the detection of emerging higher levels of structural and functional organization. In contrast with the reductionist approach, Systems Biology deals with the reconstructive and integrative task upon the available biological information. And it is here where models and modeling becomes a central tenet in Systems Biology.

In the following section we will develop a general framework where the role of models and the modeling process within the scientific activity in biosciences is highlighted. Also, a set of rules that help the modeling activity is presented together with some general considerations on the challenges that modeling currently poses.

## A Model of the Modeling Process in Biosciences

The purpose of models is not to fit the data but to sharpen the questions.

Samuel Karlin

**FIGURE 1:**
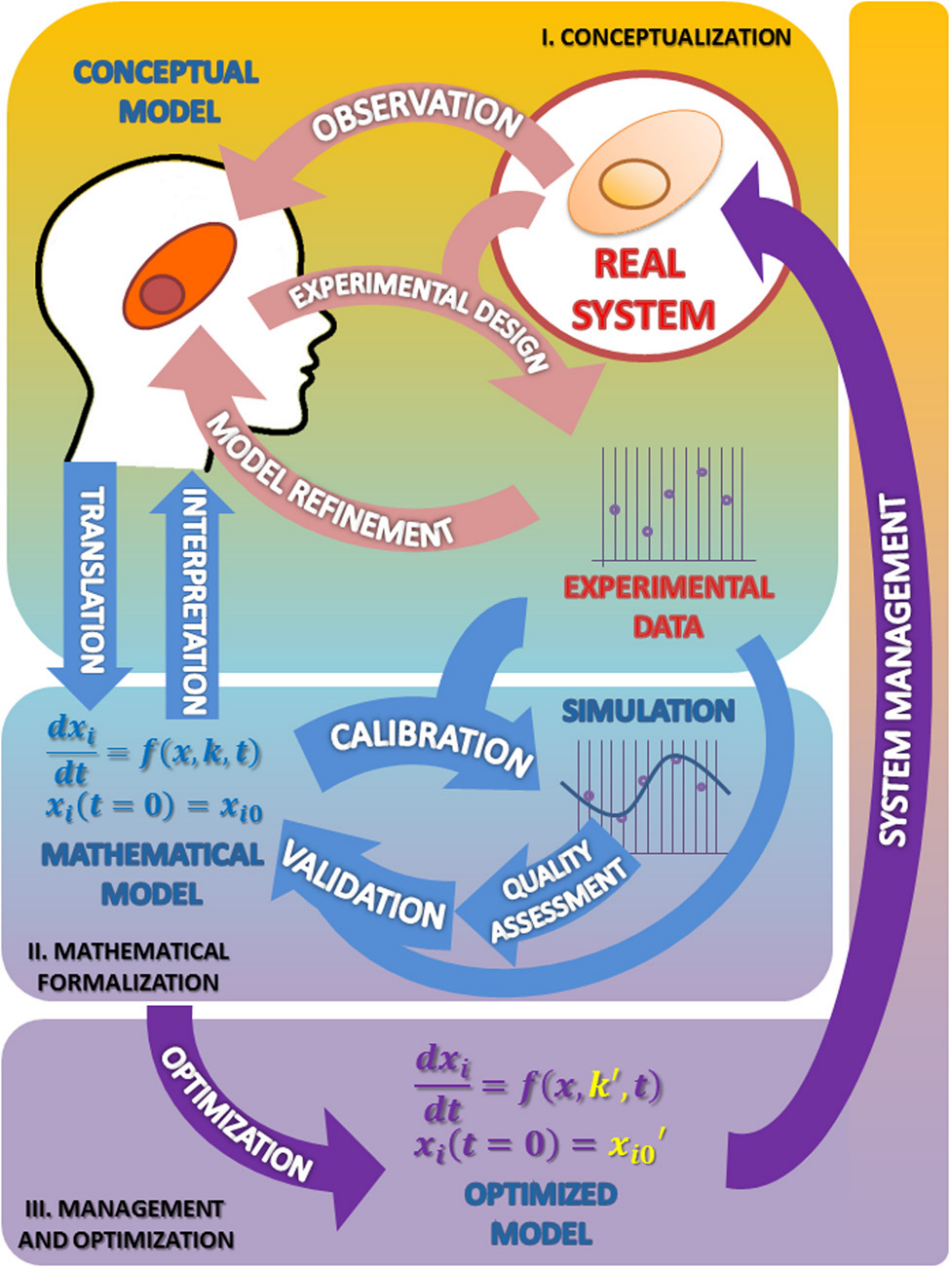
**The modeling process in biosciences**. The main activities involved in this procedure are observation followed by mathematical modeling; simulation, analysis, optimization and back to observation. In this cycle the mathematical model occupies, just after the real system, the center position. **I. Conceptualization**. Having chosen the subject of research and after some initial observations are made, the biologist should reflect on the model to be built. From the information available and a set of well-founded hypothesis, it will build a first version of the model that presents a first selection of variables, processes and interactions considered relevant (conceptual model). The iteration of this process constitutes the classical version of the scientific method (light pink arrows). **II. Mathematical formalization.** From this proposal the first mathematical formulation of the model is derived (Mathematical model). Getting to this point has required an exercise of integration of hypotheses and information that yields a new, deeper degree of knowledge about the system not reached before (light blue arrows). **III. Management and optimization.** As a result of these two phases the information needed to validate the model becomes evident, which in turn suggests new experimental designs that propitiate a new round of improvement cycle (purple arrows). As can be seen the process of building a model, itself determines the path to a greater and coherent understanding of the system that makes feasible its rational control and management. See text for more information.

The **Figure 1** summarizes the set of activities and elements involved in the development of models, as organized following the Scientific Method.

### I. Conceptualization

The first stage of the scientific modeling process is the conceptualization phase. In any research process all activities are organized around the Real System, which is the compulsory, continuous reference in the whole process. This central position is represented in **Figure 1** as a circle.

The first step in the conceptualization stage is to formulate, from the very first observations of the phenomenon (Observation; see **Figure 1**), generally made in an unsystematic form, an explanatory hypothesis of it: the first version of the conceptual model. This is a critical task where it is necessary to coordinate, to contrast and discuss many issues with the aim of making the best decisions. Some of the questions that should be addressed at this stage are: what aspects of the real system should be incorporated into the model? What features should/can be ignored? Or, what hypotheses can support the observations/information rendered by the system?

Given that any model is an instrument designed for a purpose, the very first question that should be posed at this stage is: what is the model for? That is, the objective of the model. No model makes sense or is justified for its own sake. Thus, what first defines a model is the specific question that it is going to answer.

Trying to develop a model to explain all aspects of a biological phenomenon will be practically impossible, a very complex and highly unmanageable task. However, a model with a limited purpose will be feasible, and easier to be analyzed and managed. At this stage of modeling, our thinking process uses the categories of space, time, substance (namely, material components, and elements), quality, quantity, and relationship. These categories help us to bring order to the perceived complexity of the real world. Nevertheless, this act of classification and identification differ considerably from one scientific discipline to another.

The meaning and significance of the modeling process is rooted in the core of the scientific process: from the observation of some part of the biological world some questions arise, the model being the tool that eventually would serve to provide an answer. As can be seen, any modeling exercise forces, from the very beginning, to define and make explicit the focus of our research and to keep, all along the way, our attention on the main objective.

The conceptualization stage is where modeling becomes very often an art, a subjective task. The choice of the essential attributes of the real system and the omission of irrelevant ones requires a selective perception that you cannot specify through an algorithm. There is some dosage of freedom and arbitrariness at this stage since different researchers equally well informed can define different models. As we are educated in a specific biological scientific discipline, we are trained to observe the real world in the light of a certain conceptual framework.

In some instances, the discussion of contrasting opinions addressed to demarcate the border between the system and its environment, or to discriminate between different possible scenarios or to evaluate the importance of the experimental error associated with the observed values, leads to different versions of the model. Based on the final selection of hypotheses, the next step is to carry out experiments (Experimental design; see **Figure 1**) devised to obtain experimental data to test the chosen hypothesis. From the analysis of the experimental results, the hypothesis can be reformulated or discarded (Model refinement; see **Figure 1**), thereby initiating a virtuous cycle (pink arrows) that leads to an improved conceptual model. Eventually, this refined model version is expected to answer, though qualitatively, the questions initially raised. At this stage, the need to change the initial hypothesis, far from being a failure, should be understood as progress toward a better understanding of the behavior of the system. This allows to rule out some proposals, which will be replaced by new ones that might be more effective in the building process of the conceptual model.

The above sequence illustrates the fact that observation and science are not the same thing. The aim of the scientific method is not to describe but to explain the observed, to understand and interpret the observations. It is here where the collaboration between the modeling part and the field experts becomes essential. And it is at this stage where interdisciplinarity occurs. The best version of the modeling task results when it is a team effort, where the competences and expertise of different specialists blend. Those with the best knowledge on the particular subject should be able to communicate with the modeler. They must be able to understand each other; the expert presenting the whole picture and selecting from it the elements, interactions, processes, and values that are deemed relevant in the light of the model’s objective. At this point the modeler should translate this selection into a conceptual representation that usually takes de form of a mechanistic picture where the elements and their relations are represented. To be useful, this picture should be explicit enough to be translated into a series of elementary steps representing the individual mechanisms. The modeler here is instrumental in defining which are the magnitudes considered as variables and which are not; this is a critical distinction that determines to a great extent the model’s output.

The development of the modeling approach has at this point one of its great challenges, because it requires that the different specialists share a common language. There is a need, on the side of the modeler, to become acquainted with the features and nuances of the system under scrutiny, and to speak in terms easy to understand by the non-modeler party. On the other side, the specialist should adopt an integrative way of thinking and be able to make explicit his knowledge and express it in the most precise terms.

More often than not, it is necessary to repeat the conceptualization stage of discussion and analysis several times, before the proposed model becomes able to respond successfully to all the objections that could be raised by the experts who come in contact with the model. Once you have reached an acceptable version you will be able to consider the next stage: the mathematical formalization.

At this phase of the model building process it could happen that the modeler may be tempted by the challenge of building a wholly comprehensive model system, that is, one that takes into account, if not all, most of the characteristics of the real system. Besides the misunderstanding of the modeling process that this shows, this attitude has additional costs, because if two models serve to give the desired answers, the simpler one is better. A modeler intending to include all variables and parameters described would also be faced with the task of analyzing the influence of all the parameters on all the variables. This in turn would require an additional, usually non-negligible effort for its interpretation, making the model more difficult to understand. In modeling, more and harder is not necessarily better. In fact it sometimes happens that the largest and most complicated model may be the poorest in attaining its objectives or expressing necessary or meaningful details of the reality. A nice illustration of this point is the very simple model of the signaling pathway of NF-κβ, in which with only three elements it is reproduced the main dynamical behavior of the original system (Krishna et al., 2006). In other words, we should try to make the complex as uncomplicated as possible. Despite this, the discussion of its results can enrich the conceptual model building when considering the traits and characteristics that were not initially included.

Related with this is the fact that the developments of the conceptual model force the analysis and the systematic review of available knowledge about the system and its behavior. As a result of this exercise of verbalization of the knowledge -often unconscious- that experts have about the system, a new light is shed on the phenomenon, which very often contributes to a better understanding of the system.

It may happen that some gaps of information about interactions or relevant parts that had hitherto gone unnoticed become evident. This usually suggests new avenues of exploration and ultimately contributes to a better understanding of the observed reality. Also, the discussions on the variables or the processes involved help to change previous assumptions or facilitates a new view and understanding of some facts that previously remained without an explanation. As an example, Cheong et al. (2008) review the contributions of mathematical modeling on the understanding of the NF-κβ pathway. It is also very common to become aware of contradictions in the understanding of biological mechanisms. Most of the knowledge or information about an issue may pass through several authors undisputed, but when all this is mathematically formalized, problems to join all in a single framework emerge. Mathematical thinking forces to reconsider every piece of knowledge.

Finally, there is a modeling principle that should be commented here: “If the hypotheses of the model are erroneous, the conclusions raised from it will be wrong too.” As obvious as it may be, this principle is not less important. This principle should be taken into account all along the model-building process, particularly in the mathematical formalization that follows, because the resulting model should be faithful to the proposed hypothesis.

### II. The Mathematical Formalization

#### Mathematical Translation

The first question to be addressed in this new phase is about which mathematical formalism is best suited to represent the system (Translation; see **Figure 1**). There are many formal modeling approaches, based on differential equations, Bayesian equations, stochastic systems, agent-based modeling, etc. (for a review, see ElKalaawy and Wassal, 2015). Each of these has unique strengths and limitations. The choice heavily depends on the nature of the model. It often happens that a research group ends up enslaved by the modeling techniques which it dominates or prefers. For example, a team with experience in modeling using differential equations may tend to approach every problem from the standpoint of this technique, when in fact not all biological problems are deterministic. It is natural to preferentially use the methods that are best known and previously proven fruitful. But the ideal attitude is to adapt the specific modeling technique to the nature of the problem.

The task of developing a model is a process of approximation due to the simplifications that must be introduced. These simplifications should make sense in terms of the physical–chemical processes being studied, but must also be valid form a mathematical point of view. The general approach to the mathematical formulation usually involves the definition of the key variables and the expression of their functional relationship with the other variables of the system. Equations are then derived establishing the actual mathematical relationship among the variables. This derivation can be done empirically (data-driven), through the use of statistical methods (curve fitting) analytically o numerically, or by deriving the equations from theoretical considerations (model-driven). A classic example of model-driven is given by Michaelis and Menten (1913) kinetics. Other common techniques of data-driven modeling are shown in Janes and Yaffe (2006). In the model we should make clear the differences among the variables (concentrations of biochemical compounds of the investigated network: metabolites, proteins, messenger RNAs, etc.) and the parameters. Variables can be dependent, being the elements which vary over time according to the state of the system (also called states); and independent, being the ones that can be controlled during the experiment (light, pH, etc.). The parameters set internal and external constraints on the system. The specific numerical values for the parameters are determined using prior biological knowledge, such as information about the basal steady states of the system (Voit, 2000), or experimental data from dynamical perturbations (Vera et al., 2007, 2008). Usually the models integrate kinetic data and other available information about the elements of the process, as well as fluxes obtained from experimental observations.

It often happens that an existing model is used to describe another system. This strategy, although tempting, should be used with caution. Each new system should be studied in their specific conditions of environment and structure. It is also necessary to consider that a model not only depends on the system that it represents and the techniques used for its construction, but also from the motivations and objectives of their creators. Therefore one must always beware from attributing the motivations and objectives of others to our own model.

The process of developing a mathematical formulation of the conceptual model forces the investigator to describe the system in simple terms. At this stage the research team must take into account details about the system which might otherwise go unnoticed, which contribute to the improvement of the model. Also, a healthy consequence of the formalization process is that the explanations of the initial, sometimes unexamined assumptions reveal processes and features that remained unrecognized under the less precise conceptual formulations.

The interpretation and understanding of the system has an additional resource in the mathematical expression of it (see **Figure 1**). The set of equations of the mathematical model is likely to be discussed with the plethora of techniques and mathematical tools that allow the description and analysis of the complex interrelated processes that occur in the real system; these techniques can help to elucidate the structure, properties, and dynamic behavior of the system. These analyses can reveal details about the behavior of a model such as the occurrence of oscillations or other complex behaviors that are often the motivating force for studying these systems.

#### Parameter Estimation and Quality Assessment

Once the conceptual model has been translated to its mathematical form, the model should be provided with the values of its parameters. Parameter estimation or model calibration is a recurrent issue in the model building process; it deals with the finding of the numerical values which characterize the mathematical representation of a given system from experimental data (Park et al., 1997). A key feature of these experimental measurements is that they must come from variables representing their main features both at a given particular time, as well as along its evolution over time (Polisety and Voit, 2006; van Riel, 2006; Ashyraliyev et al., 2008; Banga, 2008). In addition, the quality of the model should be tested through some numerical quality assessments. The quality assessment of the model includes the evaluation of aspects such as the stability of steady states, a prerequisite for any model describing actual biological systems; and the robustness of the model, a test to evaluate whether the model is able to tolerate small structural changes (Savageau, 1971; Hsiung et al., 2008) and the dynamic features that characterize the transient responses to temporary perturbations or permanent alterations (Wu et al., 2008). These analyses often pinpoint problems of consistency and reliability of the mathematical representation (Okamoto and Savageau, 1984, 1986; Ni and Savageau, 1996a,b); this constitutes by itself a further cycle of model refinement (**Figure 1**, light blue cycle). These changes result in improvements of the initial conceptual model. The conceptual model so improved will in turn suggest further experimentation leading to a new refined version that is enriched from the formalization phase.

At all instances it should be borne in mind that both the parameters and the structure of real systems change over time. Therefore, a given model, which can be satisfactory at one time or certain conditions, may lose its effectiveness at another time or in other conditions. But the equations by themselves do not contribute much to the understanding of the system. It is necessary to solve the equations for some representative values of the parameters. Accordingly, the model is submitted to the simulation and validation processes.

#### Simulation and Prediction

The mathematical model should be programmed in the computer. The computer program is the translation of the mathematical model to another language useful for computing purposes. There are many computer languages and platforms to deal with this task; advances in computer numerical analysis have made the solution of complicated systems fast and accurate. It is at this point where computation becomes critical, since the equations describing biological processes nearly always involve control and regulatory mechanisms that have non-linear components. In contrast with linear differential equations that often can be solved analytically, non-linearities make it generally impossible. But through the use of numerical methods implemented on computers we can obtain good estimates and model predicted data.

#### Model Validation

Validation stands here as the correspondence between the real system and the mathematical model. A model can be considered good and useful only if its predictions in a given scenario agree with the experimental observations made on the actual system setting. As it is shown in the **Figure 1**, we can accept the model as a plausible representation of the system under scrutiny only when the comparison of the predicted outputs with the real ones yields similar results (and when this occurs in a significant number of situations).

The validation process can only be based on comparative observations of the output values and trajectories of the model and the real system, under certain given experimental conditions. As it is shown in the **Figure 1**, for validation purposes, a first cycle of calibration and quality assessment is needed, and then a second one, with new experimental data from a different condition. As a result, the model might require some modification in order to minimize the observed discrepancies.

There are several ways to perform the validation process. One is to compare the evolution of the variables from some, other initial conditions; with data obtained by different, other research groups in similar systems. Another way is to use all available data in some given conditions, not for the development of our own model, but to use these data for the comparison with our model’s predictions instead (Santos and Torres, 2013). In some cases, a useful technique is to vary some model’s parameters within the range of biologically plausible values, and observe how the system responds in relation to its actual behavior (Segre et al., 2002). Finally, a technique that can be used in some instances involves subjecting the model to extreme conditions, deliberately looking for their failures. The logic behind this is that, if a model represents the system well in extreme conditions, so it will under normal conditions. In any instance the observed discrepancies indicate errors in the assumptions used in the building of the mathematical and/or the conceptual model. The discrepancies may be large enough as to require the revision and change of the hypothesis of the conceptual model, or the introduction of only slight modifications in the parameters of the mathematical version.

It should be recalled that the quality of a model depends directly on the quality of the information on which it is based. This statement is just the translation to the modeling context of the classical motto: “garbage in, garbage out”. A mathematical model logically processes the information with which it has been built; it cannot recognize or correct errors in the definitions or the input information. The model predictions are the result of the assumptions used, hence the extreme importance of caring for the conceptualization phase and the quality of the initial information.

Very often the most important results of this phase are negative: a well-crafted model would serve to discard a particular mechanism as the explanation for experimental observations. After sufficient validation, we will eventually arrive to a mathematical version of the model that can be used to perform experiments in much the same manner as in the real system. A model is considered valid in this respect when the decisions made using the mathematical model are “similar” to those that would have been made by physically experimenting with the real system.

This model version and its computer counterpart allow testing conditions that may be difficult to attain in the laboratory, or that have not yet been examined in actual experiments. Each numerical solution of the model provides a simulation of a real or potential experiment carried out on the Real System. As an example of mathematical simulations which were useful to understand the dynamics of the cell membrane, a biological process elusive under laboratory experiments see Marrink and Tieleman (2013).

In this phase, starting from a first version of the mathematical model we come to an improved, validated version, through a new virtuous cycle (light blue arrows) that sum up to the first one (light pink arrows). Repeated excursion through this research loop can result in further improvements in both the mathematical and the conceptual model that provides an unbiased test of the hypothesis involved in the conceptual model. This type of feedback loops, which are an essential part of the process of developing a model (and indeed of the scientific method), must, however, stop at some point. The validation phase often leads to a situation in which a slight increase in the trust of the model requires a huge effort. In these cases, it is advisable to stop the process at this point.

#### Model Refinement and Interpretation

Once we have reached a sound mathematical version of the real system we can advance in its interpretation and understanding. At this stage, there is an opportunity to debate and criticize the validity of the consequences and results of the model. The ultimate aim should be to achieve plausible associations between the model and the real system. At this point it should be clear that, if the conceptualization process was successful, the logical conclusions are as valid as rigorous the mathematical techniques employed, given that the model’s results are a direct consequence of the hypotheses and concepts defined in the conceptualization phase.

### III. Management and Optimization

A model fulfills its objective if it is useful and fruitful for the purpose for which it was developed. The availability of such a model has then additional benefits: it allows informed management of the system and its optimization. In this vein, mathematical modeling can be combined with operations research in order to support bio-scientists in the improvement of bioprocesses with technological or biomedical purposes (Torres and Voit, 2002; Vera and Torres, 2003; Vera et al., 2010). These type of questions can be rationally answered using mathematical modeling in combination with data mining and operations research, that have been shown to be a promising approach in fields such as drug discovery (Vera et al., 2007) and operations research (Vera et al., 2010).

The optimized model, as any candidate model, should be evaluated in terms of its numerical quality in the same terms as presented above, to be a proven suitable representation of a real system (see the Parameter estimation and quality assessment section). And, as usual in these cases (see the parameter estimation and quality assessment section above) these analyses contribute to the refinement of the model through another iterative virtuous cycle (purple arrows) that superimposes to the previous one, leading to a further improved conceptual model.

## Concluding Remarks

Mathematical modeling was made possible as early as the 17th century, but it is with today’s techniques that it has become available to natural (and even social) scientists. There is already an ample evidence of the value and usefulness of the modeling approach in solving relevant problems in biosciences (Hübner et al., 2011; Lanza et al., 2012; Visser et al., 2014). However, in order to place modeling at the core of biological research it is necessary for the new generations of bio-scientists to be prepared to be able to build models. Currently, there are two conditions that must be met for this trend to accelerate. First, it is a matter of fact that the ecumenical nature of the training required by the modeling task in biosciences has impaired this desired evolution. The paradigm shift that involves the incorporation of the integrative approach requires shaping and expanding the training base of the new bioscience graduates with elements that include a broad and solid background in mathematics, thermodynamics, and scientific computing, among other new disciplines, in addition to the classic as chemistry, genetics and bioinformatics. Mathematical modeling of bioprocesses also has severe limitations for development and generalization because of the lack of training in math observed in many bioscience postgraduates (Watters and Watters, 2006; Koenig, 2011). It is our view that the best way to overcome this flaw is through the inclusion of two elements that are, at least not well developed in the curricula of the biosciences graduates, if not absent. One is the appropriate, and properly adapted mathematical contents, which could deal with the normally underdeveloped mathematical thinking of the students. There is some discussion as to what contents and to what extent the biosciences students should be exposed to (Voit and Kemp, 2009). But what seems unavoidable is the fact that the biological scientist of the XXI century should be fluent not only in mathematics (in statistics and also in other mathematical concepts such as ordinary differential equations) but also in modeling techniques. Fortunately, there is an increasing awareness in this regard and some material is already available to fill this gap (Voit, 2012; Torres, 2013).

The understanding of the system through the use of the mathematical tools that allow the description and analysis of the complex systems can help to deepen the knowledge of the structure, properties and dynamic behavior of the system. However, the collaboration with experienced mathematicians is required for analyses such as the choice of the proper numerical methods, and the selection of the valid simplifications of complicated models. This is the area where most of the typical modeling projects develop: the fertile interface among established disciplines such as cellular biology, biochemistry, genetics and mathematics, and others. In this task all parties are benefited from valuable insight from the interdisciplinary experience. Modeling implies the definition of the model’s objectives, and the curation of the available information. It facilitates not only the finding of previously unsuspected areas of exploration, but the proposition of new questions that were not at all evident from the reductionist approach. The systematic practice of modeling in this context also naturally facilitates the fusion of scientific disciplines; this unifying tension is felt not only among biological specialties (e.g., biochemistry, cell biology, microbiology, and genetics) but also with other (seemingly) distant ones, as operational research, computer science and mathematical analysis.

Most of the modelers are well between two extreme positions. On one side lie the idealistic ones who consider model building as a mental process in which the inductive dimension is not valued. For them the mathematical structure obtained represents reality. Opposed to this is the one with a pragmatic view, for whom the goal is to adjust the model to the actual data but without paying attention to a better understanding of reality. The right position would be that in which the model is adjusted to the data, but reaching an understanding of the observed reality is always the aim. The optimum position of a good modeler is halfway between the most pragmatic and utilitarian view of an engineer and the more general outlook of the philosopher.

Finally, it should be noted that although the most common approach in the current biological research is the study of the design of living organisms by observing examples available in nature, there is an inductive, subsequent task that should not be forgotten. We refer to the derivation of general principles from these examples. Efforts are being carried out to gain insight into what is possible in biological design (Savageau, 1976; Alon, 2006; Salvado et al., 2011; Wolkenhauer and Green, 2013) that may lead to practical techniques for generating designs for biological systems intended to carry out particular tasks.

## Author Contributions

ND has contributed to the conception and design of the work and the analysis and interpretation of the relevant information and the drafting and revision of it’s content. Also gave the final approval of the version to be published.

GS has contributed to the conception and design of the work and the analysis and interpretation of the relevant information and the drafting and revision of it’s content. Also gave the final approval of the version to be published.

## Conflict of Interest Statement

The authors declare that the research was conducted in the absence of any commercial or financial relationships that could be construed as a potential conflict of interest. The reviewer Ester Vilaprinyo and handling Editor Rui Alves declare that despite their previous collaborations the review process was conducted objectively.
